# Unveiling the hidden dangers: a review of non-apoptotic programmed cell death in anesthetic-induced developmental neurotoxicity

**DOI:** 10.1007/s10565-024-09895-0

**Published:** 2024-08-02

**Authors:** Haiyan Sun, Yisi Shan, Liyan Cao, Xiping Wu, Jiangdong Chen, Rong Yuan, Min Qian

**Affiliations:** 1 Translational Medical Innovation Center, Zhangjiagang TCM Hospital Affiliated to Nanjing University of Chinese Medicine, Zhangjiagang, Jiangsu China; 2 Department of Anesthesiology, Zhangjiagang TCM Hospital Affiliated to Nanjing University of Chinese Medicine, Zhangjiagang, Jiangsu China; 3 Department of Neurology, Zhangjiagang TCM Hospital Affiliated to Nanjing University of Chinese Medicine, Zhangjiagang, Jiangsu China; 4 Department of Anesthesiology, People’s Hospital of Ningxia Hui Autonomous Region, Yinchuan, Ningxia China

**Keywords:** Anesthetic-induced developmental neurotoxicity, Programmed cell death, Necroptosis, Pyroptosis, Ferroptosis, Parthanatos

## Abstract

Anesthetic-induced developmental neurotoxicity (AIDN) can arise due to various factors, among which aberrant nerve cell death is a prominent risk factor. Animal studies have reported that repeated or prolonged anesthetic exposure can cause significant neuroapoptosis in the developing brain. Lately, non-apoptotic programmed cell deaths (PCDs), characterized by inflammation and oxidative stress, have gained increasing attention. Substantial evidence suggests that non-apoptotic PCDs are essential for neuronal cell death in AIDN compared to apoptosis. This article examines relevant publications in the PubMed database until April 2024. Only original articles in English that investigated the potential manifestations of non-apoptotic PCD in AIDN were analysed. Specifically, it investigates necroptosis, pyroptosis, ferroptosis, and parthanatos, elucidating the signaling mechanisms associated with each form. Furthermore, this study explores the potential relevance of these non-apoptotic PCDs pathways to the pathological mechanisms underlying AIDN, drawing upon their distinctive characteristics. Despite the considerable challenges involved in translating fundamental scientific knowledge into clinical therapeutic interventions, this comprehensive review offers a theoretical foundation for developing innovative preventive and treatment strategies targeting non-apoptotic PCDs in the context of AIDN.

## Introduction

Due to the large global population and China’s three-child policy, the number of infants and children undergoing anesthesia and surgery has increased, and whether anesthetics are neurotoxic to children, particularly infants, has been a major concern in recent years (Ing et al. 2022; McCann and Soriano 2019).The crucial periods of rodents’ brain development occur within the first 1 to 3 weeks of life, while in humans, these stages happen between the final trimester of pregnancy and the initial 2 to 3 years of life (Maloney et al. 2019; Nadarajah and Parnavelas 2002; Olney et al. 2002; Yan and Jiang 2014). During this period, the nervous system develops rapidly and is most sensitive to various external factors that influence brain development. Repeated or prolonged anesthetic exposure can cause anesthetic-induced developmental neurotoxicity (AIDN) (Jiang et al. 2023b; Useinovic et al. 2022; Wang et al. 2023a). Numerous animal studies have demonstrated that repeated general anesthesia during pregnancy and the early postnatal period can disrupt morpho-functional changes in the brain, leading to long-term behavioral abnormalities in most animal models (Chinn et al. 2023; Li et al. 2023a; She et al. 2023). However, the clinical studies of general anesthetics neurotoxicity are still controversial (O’Leary et al. 2019; Simpao et al. 2023; Walkden et al. 2020). Nonetheless, it is well known that anesthetic-induced developmental neurotoxicity is related to the duration of anesthetic exposure and dose used. Consequently, In 2016, the United States Food and Drug Administration issued a caution stating that extended or repeated exposure to general anesthetics during late pregnancy or before the age of three may have an impact on the developing brain (Olutoye et al. 2018).

Programmed cell death (PCD) is a physiological process orchestrated by genes. Each type of PCDs is crucial for organism development, and uncontrolled PCDs can cause developmental defects. Apoptosis was previously considered to be the primary cause of neurotoxicity induced by anesthetic agents (Gholami et al. 2023; Wang et al. 2023b; Zhang et al. 2023b). Numerous studies have also demonstrated that neuronal apoptosis deregulation during synaptogenesis contributes to AIDN and is regarded as a significant mechanism of AIDN (Liu et al. 2022a; Zhang et al. 2022b; Zhang et al. 2023b). Apoptotic cells undergo caspase-dependent cell death without releasing their contents extracellularly, resulting in a non-inflammatory process. However, emerging studies propose that AIDN is related to the generation and emission of inflammatory cytokines (Wali et al. 2022; Wu et al. 2022a; Zhao et al. 2023). Consequently, the inflammatory characteristics of AIDN cannot be exclusively attributed to neuronal apoptosis. With advancements in cell death mechanism research, multiple new forms of programmed cell death have gradually come to light. The Nomenclature Committee on Cell Death (NCCD) outlined 11 types of regulated cell death in 2018, with apoptosis being one of them (Galluzzi et al. 2018). Non-apoptotic cell death mechanisms present new potential targets for treating neurological diseases (Lammert et al. 2020; Li et al. 2023b; Ryan et al. 2023). Unlike apoptosis, non-apoptotic PCDs are highly related to inflammation and oxidative stress, suggesting that they may have a stronger promoting effect on AIDN than apoptosis. Therefore, whether and how these non-apoptotic PCDs participate in the occurrence and development of AIDN has received widespread attention. In this review, we discussed the latest research on the role of non-apoptotic PCDs in AIDN. Here, by summarizing and comparing the cell death pathways involved, we aimed to explore the prevention and treatment of AIDN.

## Necroptosis

### The definition and mechanism of necroptosis

Necroptosis, a distinct type of PCD, was first outlined in 2005 by Degterev and colleagues (Degterev et al. 2005). This process differs from apoptosis and does not involve the activity of caspases. Instead, it involves the action of receptor-interacting serine/threonine protein kinase 1 (RIPK1), RIPK3, and mixed lineage kinase domain-like protein (MLKL) (Wang et al. 2014). Characteristically, necroptosis initiates organellar swelling, plasma membrane disruption, and the discharge of cellular components, which subsequently trigger an inflammatory response (Purnama et al. 2023).

**Fig. 1:**
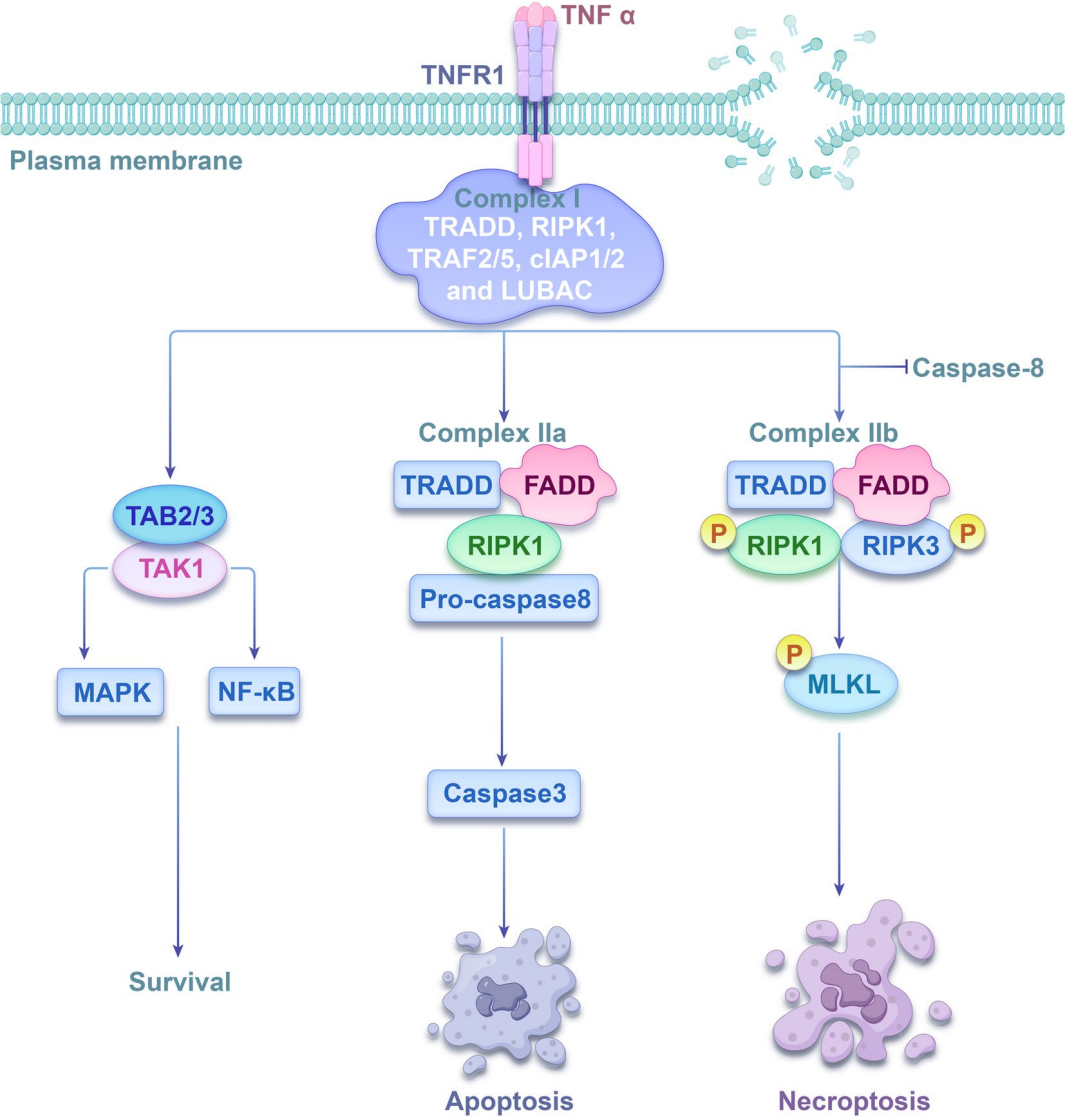
The mechanism of necroptosis

Various activators of necroptotic pathways exist, but the most extensively researched pathway causing necroptosis involves the attachment of tumor necrosis factor-α (TNF-α) to tumor necrosis factor receptor 1 (TNF-R1) (Fig. 1). Following the attachment of TNF-α to TNFR1, a cascade of downstream protein molecules is recruited to form complex I, including tumor necrosis factor (TNF) receptor-associated factor 2/5 (TRAF2/5), TNFR1-associated death domain protein (TRADD), cellular inhibitor of apoptosis protein 1 and 2 (cIAP1/2), linear ubiquitin chain assembly complex (LUBAC) and RIPK1. Within complex 1, RIPK1 can undergo ubiquitination by cIAP1/2 and LUBAC, or deubiquitination by cylindromatosis (CYLD). When RIPK1 gets polyubiquitinated, it further recruits transforming growth factor-β-activated kinase 1 (TAK1) and TAK1-binding protein 2/3 (TAB2/3), thereby activating mitogen-activated protein kinase (MAPK) and nuclear factor-kappaB (NF-κB) pathways, obstructing caspase-8 activation, and fostering cell survival (Mohanty et al. 2022; Nicolè et al. 2022; Teh et al. 2022; Xu et al. 2021). Additionally, when RIPK1 undergoes deubiquitination, TNF-α recruits TRADD, Fas-associated death domain protein (FADD), pro-caspase-8, and RIPK1, they assemble into complex IIa, which facilitates caspase-8 activation. This caspase-8 activation eventually leads to apoptosis via the caspase-3 activation (Li et al. 2022; Peng et al. 2022; Woznicki et al. 2021). If caspase-8 is inhibited or present in low levels, RIPK1 recruits RIPK3, which recruits MLKL through the receptor interacting protein (RIP) homotypic interaction motif (RHIM) to assemble complex IIb, called necrosome. Phosphorylated MLKL undergoes oligomerisation and translocation to the plasma membrane. This leads to the generation of membrane-penetrating pores which disrupt the integrity of the plasma membrane and result in necroptosis (Karlowitz and van Wijk 2023; Martens et al. 2021; Sethi et al. 2022). Notably, necroptosis can also occur without RIPK1 (RIPK1-independent necroptosis) (Jantas and Lasoń 2021). When RIPK3 concentrations increase, TNFR1 ligation communicates through TRADD to stimulate RIPK3. Interestingly, TNF can prompt the activation of RIPK3, even in scenarios where RIPK1 is absent. Once active, RIPK3 can trigger two different pathways leading to cell death, one pathway involves necroptosis mediated by MLKL, while the other pathway involves apoptosis driven by caspase 8 (Moujalled et al. 2013).

### Necroptosis role in AIDN

The regulatory mechanisms of necroptosis-associated genes in AIDN progression are shown in Table 1. Sevoflurane, an inhalational anesthetic widely used for infants and children, has been linked to developmental neurotoxicity through apoptosis and necroptosis mechanisms, as noted by Xu et al. (Xu et al. 2022). In hippocampal neurons and tissues, exposure to sevoflurane resulted in increased concentrations of RIPK1, RIPK3, and p-MLKL. Additionally, the co-immunoprecipitation findings indicated a notable increase in the protein binding of RIPK1/RIPK3 following sevoflurane exposure. The study further revealed that apoptosis inhibition causes an increase in necroptosis, which implies that necroptosis acts as an "insurance policy" when apoptosis is suppressed. However, administration of the RIPK1 activity blocker Necrostatin-1 (Nec-1) alone did not alleviate sevoflurane-induced developmental neurotoxicity, implying that RIPK1 may not be crucial for RIPK3/MLKL-mediated necroptosis in AIDN.

**Table 1:** The regulatory role of non-apoptotic PCDs related genes in AIDN progression

AIDN Models	Target	Mechanism	Type of Cell Death	Reference
sevoflurane exposure	-	RIPK1/RIPK3	necroptosis	Xu et al. 2022
sevoflurane exposure	ZBP1	RIPK3/MLKL	necroptosis	Wang et al. 2022c
sevoflurane exposure	NF-κB	Caspase-1/11	pyroptosis	Dai et al. 2021
sevoflurane exposure	HTR2A	miR-140-3p /DNMT1/HTR2A/ERK/Nrf2	pyroptosis	Wu et al. 2022b
sevoflurane exposure	PHLDA1	TRAF6/Rac1	pyroptosis	Shu and Du 2022
sevoflurane exposure	mTORC1	GSDMD	pyroptosis	Wang et al. 2023
sevoflurane exposure	HMGB1	TLR4/NF-κB	pyroptosis	Shan et al. 2023
sevoflurane exposure	SETD1B	H3K4 methylation/CXCR4	pyroptosis	Wang et al. 2024
ketamine exposure	-	NLRP3/Caspase-1	pyroptosis	Ye et al. 2018 Zhang et al. 2021
propofol exposure	HOTAIR	miR-455-3p/NLRP1	pyroptosis	Gong et al. 2021
dexmedetomidine /propofol exposure	HOXA5	NLRP3	pyroptosis	Wang and Wan 2023
Ketamine/ sevoflurane exposure	NMDAR	RASD1/ PAP7/DMT1	ferroptosis	Wu et al. 2020
sevoflurane exposure	-	ACSL4,COX2,GPX4,FTH1	ferroptosis	Zhang et al. 2022a
sevoflurane exposure	Nrf2	ROS	ferroptosis	Song et al. 2022
sevoflurane exposure	15LO2- PEBP1	ATM/ p53/SAT1/15LO2-PEBP1	ferroptosis	Jiang et al. 2023b
sevoflurane exposure	ATF3	ER stress/PERK/ATF4/ATF3	ferroptosis	Kang et al. 2024
isoflurane exposure	GPX4	ROS	ferroptosis	Xia et al. 2018
isoflurane exposure	system Xc −	ETC activity (Complex IV)	ferroptosis	Liu et al. 2021
sevoflurane exposure	ROS	PARP-1	parthanatos	Piao et al. 2020
sevoflurane exposure	-	STEP61/Pyk2/ROS/ PARP-1	parthanatos	Wang et al. 2022a

Building on the finding, Wang et al. (Wang et al. 2022c) explored alternative upstream targets in the RIPK3/MLKL pathway. This investigation revealed that sevoflurane exposure during development led to necroptosis mediated by RIPK3/MLKL, resulting in plasma membrane rupture. This process promoted the generation and dissemination of inflammatory cytokines. Furthermore, Z-DNA/RNA-binding protein 1 (ZBP1) was recognized as a crucial signaling target for necroptosis (Jiao et al. 2020). This research provided evidence that ZBP1 detected cytosolic mitochondrial DNA (mtDNA) and subsequently activated the RIPK3/MLKL pathway in the scenario of developmental sevoflurane exposure. Moreover, as both RIPK1 and RIPK3 can bind to ZBP1 for mediating cell necroptosis, this study established the respective contributions of RIPK1 and RIPK3 in MLKL-mediated necroptosis through the RIPK1 or RIPK3 siRNAs transfection before sevoflurane exposure. It was observed that the phosphorylated MLKL level on the membrane was markedly reduced by RIPK3 siRNA transfection. Contrarily, the phosphorylated MLKL level in the membrane was unaffected by RIPK1 siRNA transfection. This study also observed that sevoflurane exposure significantly elevated ZBP1-RIPK3 interaction, but not ZBP1-RIPK1. These results imply that RIPK3, rather than RIPK1, is essential for MLKL-driven necroptosis initiation in AIDN. These findings suggest that inhibiting necroptosis may prevent AIDN. However, research on necroptosis and AIDN is limited. Furthermore, the activation and inhibition mechanisms of upstream and downstream factors like caspase, RIPK3, and MLKL associated with AIDN have not been thoroughly investigated. This study represents an innovative avenue for future research.

## Pyroptosis

### The definition and mechanism of pyroptosis

Zychlinsky and associates (Zychlinsky et al. 1992) first identified pyroptosis in 1992 when they observed that caspase-1 was necessary for the death of *Shigella flexneri*-infected macrophages. Later, in 2001, Cookson and Brennan (Cookson and Brennan 2001) discovered a related event in macrophages harboring *Salmonella typhimurium* infections, and they came up with the word "pyroptosis" to characterize this kind of cell death. ‘Pyroptosis‘ was defined by NCCD in 2009 (Kroemer et al. 2009) and 2012 (Galluzzi et al. 2012) as a form of inflammatory cell death caused by activation of caspase-1. NCCD revised this definition one more up until 2018 (Galluzzi et al. 2018): Pyroptosis is a kind of cell death that is dependent on gasdermin protein family creating plasma membrane pores; it is frequently (though not always) brought on by the activation of inflammatory caspases. Morphologically, proinflammatory factor release, osmotic lysis, and cell swelling are the hallmarks of pyroptosis (Yu et al. 2021).

**Fig. 2:**
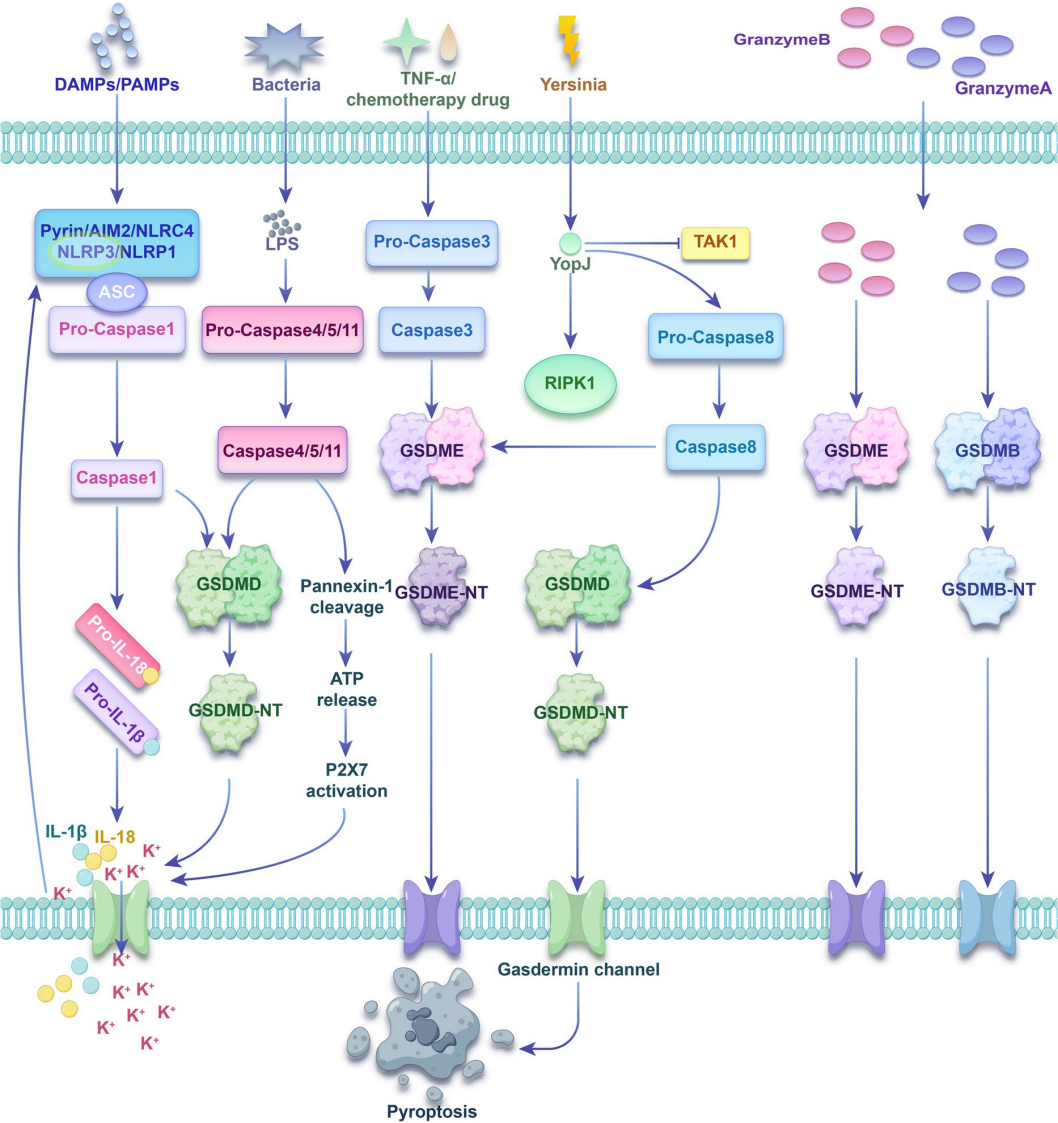
The mechanism of pyroptosis

Pyroptosis was assumed to involve two distinct pathways: a canonical one reliant on caspase-1 and a non-canonical one dependent on either human caspase-4/5 or mouse caspase-11. Nevertheless, other pyroptosis pathways have been discovered through intensive research in recent years. The mechanisms and pathways of pyroptosis are described in detail below (Fig. 2).

The canonical pyroptosis pathway is facilitated by inflammasome assembly and relies on caspase-1. Inflammasomes are usually composed of three parts: (1) receptor proteins, including NOD-like receptor protein 1 (NLRP1), NOD-like receptor protein 3 (NLRP3), NOD-like receptor C4 (NLRC4), absent in melanoma 2 (AIM2) and pyrin. NLRP3 is the most comprehensively investigated receptor protein (Lu et al. 2020); (2) The adaptor protein, apoptosis-associated speck-like protein (ASC), includes a caspase activation and recruitment domain (CARD). CARD is critical for pro-caspase-1 recruitment. Certain pattern recognition receptors, like pattern recognition receptors (PRRs), also possess CARD and can directly recruit pro-caspase-1 (Broz and Dixit 2016); (3) Pro-caspase-1 (caspase effector). Pro-caspase-1 undergoes hydrolysis to mature caspase-1 after inflammasome formation. Mature caspase-1 is capable of cleaving gasdermin D (GSDMD) into its N-terminal (GSDMD-NT) and C-terminal (GSDMD-CT) domains. Multiple GSDMD-NTs oligomerize and transport to the cell membrane, creating unselective pores that cause cell swelling and rupture. Moreover, mature caspase-1 splits pro-interleukin-1β (pro-IL-1β) and pro-interleukin-18 (pro-IL-18), forming mature IL-1β and IL-18. These are then transported outside the cell via membrane pores, leading to cell pyroptosis (Ding et al. 2023; Liao et al. 2023; Sun et al. 2023).

The non-canonical pyroptosis pathway relies on either human caspase-4/5 or mouse caspase-11, which can directly bind to bacterial lipopolysaccharide without requiring an inflammasome sensor. The activated caspase-4/5 or caspase-11 can split GSDMD, as opposed to pro-IL-1β and pro-IL-18. GSDMD-NTs are transported to the cell membrane, leading to pore generation in the plasma membrane. The membrane pores can trigger the efflux of intracellular potassium ions, thereby activating the NLRP3 inflammasome and the canonical pyroptosis pathway and eventually leading to the cleavage and release of pro-IL-1β and pro-IL-18 (Cheng et al. 2021; Drummer et al. 2023; Miao et al. 2019; Shi et al. 2014). Furthermore, caspase-4/5/11 can split pannexin-1, resulting in adenosine triphosphate (ATP) release and the opening of purinergic ligand-gated ion channel 7 (P2X7) receptor channels. This, in turn, results in potassium efflux, NLRP3 inflammasome activation, and the production and efflux of IL-1β and IL-18 (Yang et al. 2015).

Besides the above two pathways, pyroptosis can also be triggered by some apoptotic caspases. Historically, caspase-3 has been viewed as a key indicator and pivotal component in apoptosis. However, recent studies have demonstrated its role in pyroptosis. In response to activators like TNF-α or chemotherapeutic agents, gasdermin E (GSDME) is cleaved by caspase-3 rather than GSDMD. Multiple gasdermin E-N-terminals (GSDME-NTs) oligomerize and transport to the cell membrane, generating unselective pores that cause pyroptosis (Wang et al. 2017; Wang et al. 2018). Remarkably, activated caspase-3 can cause pyroptosis with a high GSDME, whereas a low GSDME promotes apoptosis (Zhai et al. 2022). Furthermore, caspase-8 can also act as pyroptosis inducer. YopJ, the effector protein of pathogenic Yersinia, inhibits TAK1 in mouse macrophages, which in turn activates RIPK1 and caspase-8. The activated caspase-8 then cleaves GSDMD and GSDME, leading to the formation of plasma membrane pores. These pores mediate pyroptosis and trigger an inflammatory response (Orning et al. 2018; Sarhan et al. 2018). In 2021, a study in *Science* found that the Rag-Ragulator complex (containing RagA, RagC, and Lamtor1-5) was involved in regulating pyroptosis induced by Yersinia infection (Zheng et al. 2021). The validation results showed that the Rag-Ragulator complex specifically participated in toll-like receptor (TLR)/TNF-caspase-8-GSDMD pathway-mediated pyroptosis but not in canonical or non-canonical inflammasome-mediated pyroptosis.

Moreover, a 2020 study (Zhang et al. 2020) published in *Nature* found that granzyme B of Killer Cells can activate target cells to undergo caspase-independent pyroptosis by directly cleaving GSDME, which has an identical site as caspase-3. In the same year, *Science* (Zhou et al. 2020) reported that cytotoxic lymphocyte-secreted granzyme A can release gasdermin B-N-terminal (GSDMB-NT) fragments by cleavage of gasdermin B (GSDMB), causing cell perforation and inducing pyrolysis. This finding redefines cellular pyroptosis and rewrites the conclusion that pyroptosis can exclusively be activated through caspases.

### Pyroptosis role in AIDN

The regulatory mechanisms of pyroptosis-associated genes in AIDN progression are shown in Table 1. Sevoflurane is a commonly used inhalational anesthetic for infants and children. A 2021 study (Dai et al. 2021) initially disclosed that repeated exposure to sevoflurane elevated the levels of NLRP3, caspase-11, caspase-1, and NF-κB, leading to neuroinflammation and pyroptosis in the hippocampus of developing rats. Notably, pretreatment with the selective NF-κB blocker BAY 11–7082 blocked both canonical and non-canonical pyroptotic pathways. Additionally, BAY 11–7082 improved long-term cognitive function and attenuated hippocampal neuronal injury and synaptic malfunction in adolescent rats following early sevoflurane exposure. These findings indicate that pyroptosis, mediated by NF-κB, may play a significant role in cognitive deficits induced by sevoflurane in the developing brain. NF-κB inhibition may be a strategy to ameliorate anesthesia-induced neuroinflammation and developmental neurocognitive dysfunction.

Furthermore, Wu et al. (Wu et al. 2022b) report that neonatal rats exposed to sevoflurane showed decreased expression of 5-hydroxytryptamine receptor 2A (HTR2A) in their hippocampus tissue. Moreover, it was observed that sevoflurane exposure notably elevated the levels of proteins related to pyroptosis, along with the pro-apoptotic proteins Bax and Cleaved caspase-3. Conversely, anti-apoptotic protein B cell lymphoma 2 (BCL-2) downregulation was observed in hippocampal neurons of rats. This impact was further amplified by subsequent sh-HTR2A therapy, but it was reversed by additional oe-HTR2A treatment. In addition, HTR2A is essential for developing neurological disorders. Both adult cognitive performance and infant brain development can be altered by its epigenetic modulation through DNA methylation (Paquette and Marsit 2014). Further investigation of the downstream and upstream mechanisms of HTR2A revealed that miR-140-3p may inversely target DNA methyltransferase-1 (DNMT1) to repress HTR2A promoter methylation and enhance its transcription. This subsequently activates the extracellular-signal-regulated kinase (ERK)/nuclear factor erythroid 2-related factor 2 (Nrf2) pathway, contributing to the reduction of cognitive impairment in rats. The results imply that miR-140-3p identification is a novel controller of sevoflurane induced cognitive impairment and provides a new molecular perspective for the formulation of therapies for cognitive dysfunction.

Continuing this line of research, the crucial role of epigenetic regulation in the context of AIDN was underscored in a recent study (Wang et al. 2024). The histone methyltransferase, SET domain containing 1b (SETD1B), is implicated in several neurodevelopment-related biological processes (Weerts et al. 2021). A deficiency in SETD1B could potentially lead to a range of neurodevelopmental disorders, including developmental delay, intellectual disability, seizures, and behaviors that resemble autism (Hiraide et al. 2018; Michurina et al. 2022). The study discovered that sevoflurane could lower the level of C-X-C motif chemokine receptor 4 (CXCR4) by inhibiting the SETD1B-mediated histone 3 lysine 4 (H3K4) methylation modification, thereby intensifying neuronal pyroptosis instigated by the NLRP1/Caspase1 pathway, which in turn leads to cognitive dysfunction in neonatal mice (Wang et al. 2024). Consequently, the epigenetic regulatory mechanism of SETD1B in sevoflurane developmental neurotoxicity offers a new target for treating neonatal cognitive impairment.

Moreover, having an in-depth insight into the molecular and cellular mechanisms of pyroptosis in AIDN may be valuable for drug target screening. According to Shu et al. (Shu and Du 2022), prolonged sevoflurane exposure in neonatal rats led to hippocampal impairment and neuron loss. Meanwhile, the level of pleckstrin homology-like domain family A member 1 (PHLDA1) was elevated in sevoflurane-exposed rats hippocampus. PHLDA1 is linked to inflammation, oxidative stress, and neuronal apoptosis in ischemic stroke (Liu et al. 2023a; Zhao et al. 2022). Silencing PHLDA1 relieved pathological alterations in the hippocampus and increased the number of hippocampal neurons in rats treated with sevoflurane. Furthermore, PHLDA1 knockdown reduced sevoflurane-induced neuronal apoptosis and also mitigated the levels of proteins related to pyroptosis. Further research into the downstream mechanisms of PHLDA1 unveiled that the neuroprotective effect of PHLDA1 deficiency against sevoflurane exposure in developing brains was linked to tumor necrosis factor receptor associated factor-6 (TRAF6)-mediated ubiquitination of ras-related C3 botulinum toxin substrate 1 (Rac1). The results demonstrate that blocking PHLDA1 may potentially be a therapeutic option for managing sevoflurane-induced neurotoxicity in pediatric patients.

Lastly, according to Wang et al. (Wang et al. 2023a), sevoflurane-induced developmental neurotoxicity was associated with aberrant activation of the mammalian target of rapamycin complex 1 (mTORC1) rather than the mammalian target of rapamycin complex 2 (mTORC2). czThese complexes perform distinct functions within the cell. mTORC1 has traditionally been involved in integrating nutrient and environmental information to regulate the balance between anabolism and catabolism. Meanwhile, mTORC2 is vital in cytoskeletal behavior and various pro-survival signaling pathways (Liu and Sabatini 2020). Deregulated mTOR signaling in the developing brain has previously been found to be linked to synaptic pathology and cognitive impairments (Pagani et al. 2021; Tang et al. 2014). In model of sevoflurane-induced developmental neurotoxicity, downregulation of the mTORC1 component Raptor reduced the release of damage-associated molecular patterns (DAMPs) and subsequent plasma membrane rupture (PMR). The mTORC2 component Rictor’s downregulation, however, did not have the same result. Furthermore, the activation of mTORC1 requires the involvement of several Ras-related small GTPases (Zhu and Wang 2020). Thus, this study discovered that mTOR signaling inhibition or blocking the expression of Ras-related small GTPases RagA or RagC reversed sevoflurane-induced pyroptosis. Additionally, pyroptosis inhibitors (disulfiram (DSF) or necrosulfonamide (NSA)) injection inhibited GSDMD pore formation, reduced developmental sevoflurane exposure-induced DAMPs release and subsequent PMR. Pyroptosis inhibitors also improved spatial and emotional cognitive impairment in rats without affecting locomotor activity. The data imply that mTORC1-dependent and GSDMD-mediated pyroptosis plays a promotive role in AIDN. Contrarily, DSF and NSA, being clinically approved medicines, present promising therapeutic options for preventing sevoflurane-induced developmental neurotoxicity.

Building upon these findings, Studies on sevoflurane-induced developmental neurotoxicity in rodents have focused on postnatal day 7 (P7), a pivotal synaptogenesis phase. Recent emerging literature suggests that maternal anesthesia in rodents also has adverse impacts on the offspring. According to Shan et al. (Shan et al. 2023), exposure to sevoflurane during the third trimester adversely affected long-term cognitive function in rat offspring, with NLRP3/ASC inflammasome activation, microglial activation, and high-mobility group box 1 (HMGB1) upregulation in the hippocampus and cerebral cortex. HMGB1, known as a DAMP molecule in the brain, can activate TLR 2 and 4 and consequently trigger an inflammatory response when released (Ren et al. 2023). Research revealed that HMGB1 improved NLRP3 inflammasome formation, potentially initiating the pyroptosis pathway. Administering glycyrrhizin, an HMGB1 blocker, successfully prevented cognitive deficits and inhibited NLRP3/ASC inflammasome and microglial activation in rat offspring. In conclusion, blocking HMGB1 may prevent sevoflurane-induced neurotoxicity in offspring. Further research into the downstream mechanisms of HMGB1 revealed that elevated HMGB1 regulated NLRP3/ASC inflammasome activation through the TLR4 pathway. It is noteworthy that the PI3K/Akt/mTOR signaling pathways, which are integral to cell survival and growth, may also play a significant role in modulating neuroinflammation and neurotoxicity. A study by Kamranian et al. (Kamranian et al. 2023) demonstrated the neuroprotective potential of trimetazidine against tramadol-induced neurotoxicity through the activation of these pathways. The Akt pathway, in particular, has been implicated in the regulation of inflammation and cell death, suggesting that it may be a common target for neuroprotection against various neurotoxicants, including anesthetics like sevoflurane. While the study by Kamranian et al. focused on tramadol, the implications of their findings could extend to other neurotoxic agents, potentially highlighting the Akt pathway as a key modulator in the neurotoxicity associated with anesthetics. However, the study by Shan et al. (Shan et al. 2023) was limited as it did not use any targeted inhibition or activation of the TLR4/NF-κB pathway to explore the precise role of this signal in sevoflurane neurotoxicity. Nonetheless, previous reports suggest that NF-κB activation and subsequent release of proinflammatory cytokines is the underlying mechanism for sevoflurane-induced neurotoxicity. Consequently, targeting HMGB1 or NLRP3 inflammasomes may be an efficacious intervention to counteract the neurotoxic impact of sevoflurane in offspring. Investigating these mechanisms may provide researchers with fresh perspectives on pyroptosis’s role in sevoflurane-induced developmental neurotoxicity.

Furthermore, ketamine is a noncompetitive antagonist of N-methyl-D-aspartate (NMDA) and is widely accessible for use in pediatrics as an intravenous anesthetic. It is increasingly evident that ketamine may induce neuronal death in developing brains (Huang et al. 2024; Liu et al. 2013). The necessity of caspase-1-mediated pyroptosis for ketamine-induced developmental neurotoxicity was first revealed by a study in 2018 (Ye et al. 2018). The investigation revealed that active caspase-3 and caspase-9, which are related to cytochrome C release, and mitochondrial translocation of p53, which is linked to mitochondrial apoptosis, were markedly elevated in ketamine-induced hippocampal neurotoxicity. Multiple ketamine doses significantly increased pyroptosis-related protein levels. In addition, silencing caspase-1 markedly suppressed pyroptosis-associated proteins and mitochondrial apoptosis-related proteins in an in vitro cell model. Meanwhile, the translocation of caspase-1 and NLRP3 to the mitochondria was diminished, resulting in a decrease in the formation of the NLRP3 and caspase-1 complex within the mitochondria of primary hippocampal neurons. The findings suggest that NLRP3/caspase-1 complex recruitment to mitochondria participates in the crosstalk between apoptosis and pyroptosis and may serve as a new molecular target for ketamine-induced apoptosis. According to a recent study (Zhang et al. 2021), ketamine-induced developmental neurotoxicity and cognitive deficits are also related to NLRP3/caspase-1 axis-mediated pyroptosis. They revealed that MCC950, an NLRP3 blocker, or VX-765, a caspase-1 blocker, could mitigate pyroptosis, lessen neuropathological damage, and alleviate cognitive deficits. The findings imply that the use of MCC950 or VX765 to hinder the NLRP3/caspase-1 pathway could potentially alleviate ketamine-induced cognitive decline and neurotoxicity in developing rats. In this context, the work of Mobinhosseini et al. (Mobinhosseini et al. 2024) is particularly noteworthy. They demonstrated that the innovative use of nanoparticles to deliver curcumin and zinc oxide could provide a novel approach to counteract the neurotoxic effects of ketamine. The findings underscore the potential of nanotechnology in developing neuroprotective agents that can intervene in the molecular pathways triggered by anesthetic agents, offering a valuable addition to the ongoing research in this field.

Additionally, propofol is another type of intravenous anesthetic that is commonly employed in pediatrics to induce and maintain anesthesia during surgery. However, excessive propofol use may trigger neurotoxicity (Liang et al. 2021; She et al. 2023), leading to cognitive impairment in brain development, as per current research. Gong et al. (Gong et al. 2021) demonstrated that propofol dose-dependently inhibited hippocampal neuronal viability, resulting in neuronal death. Concurrently, mRNA levels for the long non-coding RNA HOX transcript antisense RNA (HOTAIR) escalated dose-dependently in propofol-treated neurons. Studies have demonstrated the regulatory role of HOTAIR in nervous system disorders (Wang et al. 2022b). HOTAIR silencing by shRNA (sh-HOTAIR) in propofol-treated primary hippocampal neurons significantly increased neuronal viability and inhibited neuronal pyroptosis. Further investigation into the underlying mechanisms of HOTAIR uncovered its role as an adsorption sponge for miR-455-3p, which in turn targets NLRP1, diminishing its expression. Moreover, NLRP1 overexpression greatly counteracted the promotional impact of shHOTAIR on neuronal viability and reversed its inhibitory impact on neuronal pyroptosis. These outcomes suggest that HOTAIR may serve as a potential target for the treatment of neurotoxicity caused by propofol. A study in 2023 (Wang and Wan 2023) demonstrated that propofol reduced neuronal viability dose-dependently again, while pretreatment with the α2-adrenoceptor agonist dexmedetomidine improved the viability of hippocampal neurons and inhibited propofol-induced pyroptosis of hippocampal neurons. Moreover, hippocampus neurons exposed to propofol showed upregulated homeobox A5 (HOXA5) expression, while those treated with dexmedetomidine showed downregulated expression of HOXA5. Meanwhile, HOXA5 overexpression reversed the inhibitory effect of dexmedetomidine on propofol-induced pyroptosis in hippocampal neurons. HOXA5 is defined as a developmental transcription factor associated with embryogenesis and can directly bind to the promoter regions of its downstream genes to increase their expression (Jing et al. 2021). Subsequent research on the downstream mechanisms of HOXA5 revealed that HOXA5 binding to the NLRP3 promoter promoted NLRP3 expression. Furthermore, NLRP3 overexpression averted dexmedetomidine pretreatment-induced repression of propofol-induced pyroptosis in the hippocampal neurons. These results indicate that dexmedetomidine pretreatment represses NLRP3 expression by limiting HOXA5 expression, which mitigates propofol-induced pyroptosis in hippocampal neurons. This may broaden our understanding of propofol and dexmedetomidine and provide a promising approach for mitigating propofol-induced pyroptosis in hippocampal neurons. Nevertheless, these molecular mechanisms have only been explored in vitro. Animal and clinical investigations are needed to confirm propofol’s developmental neurotoxicity.

## Ferroptosis

### The definition and mechanism of ferroptosis

In 2003, Dolma et al. (Dolma et al. 2003) observed that erastin-induced cell death did not alter nuclear morphology, DNA fragmentation, or caspase 3 activations. Additionally, caspase blockers were unable to rescue this process. Afterwards, Yagoda (Yagoda et al. 2007) and Yang (Yang and Stockwell 2008) et al. discovered that this type of cell death could be suppressed by iron chelators. Furthermore, they identified another compound, RAS-selective lethal 3 (RSL3), that can induce this form of cell death. It was not until 2012 that this type of non-apoptotic PCD was officially recognized as ferroptosis (Dixon et al. 2012). Ferroptosis is characterized morphologically by burst outer membranes, normal-sized nuclei, decreased or nonexistent mitochondrial cristae, and condensed mitochondria with heightened membrane density.

**Fig. 3:**
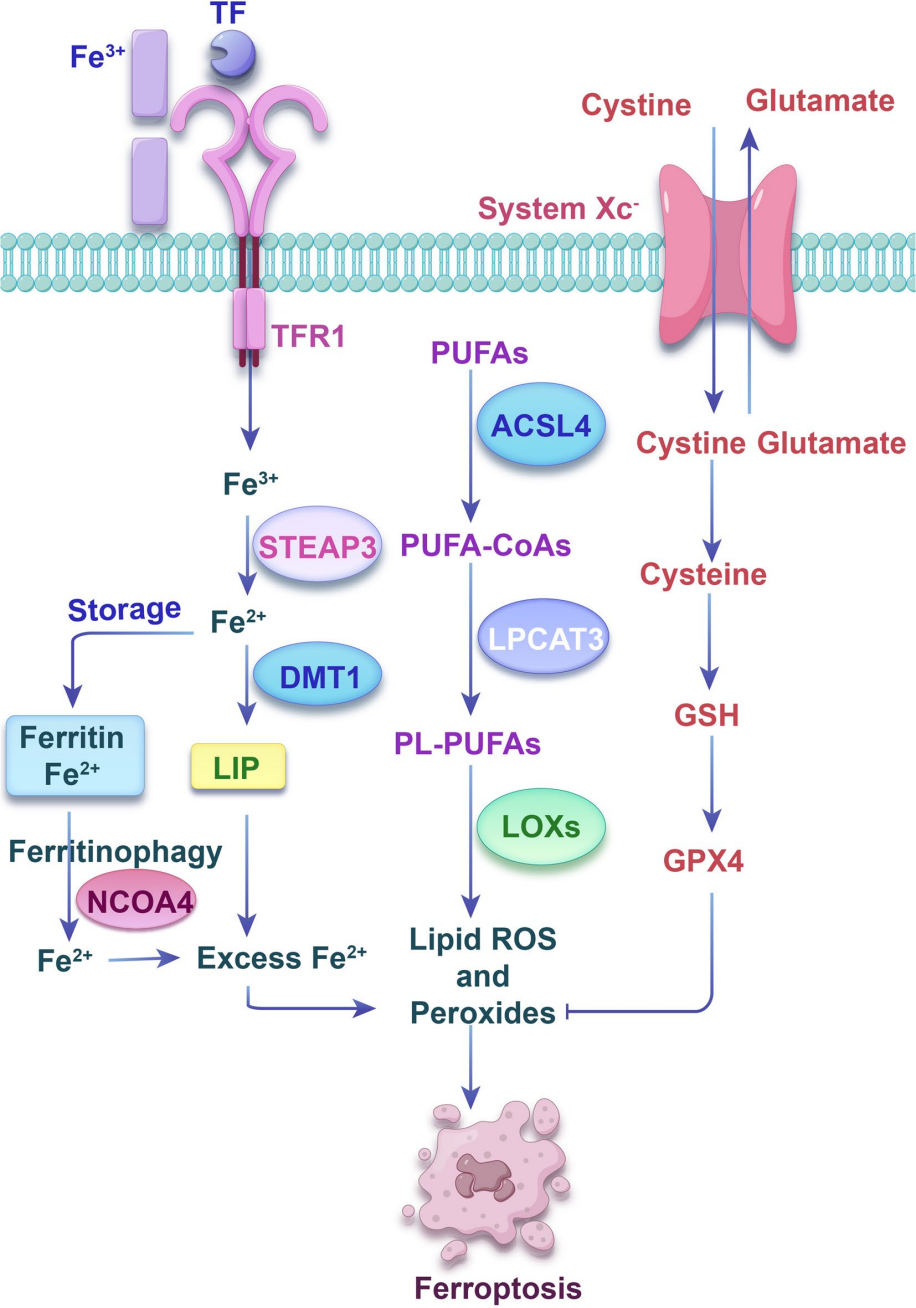
The mechanism of ferroptosis

The current regulatory mechanism of ferroptosis involves multiple metabolic networks and signaling pathways (Dixon and Pratt 2023). It is primarily linked to iron metabolism disorders, imbalance of the amino acid antioxidant system, and lipid peroxide accumulation (Fig. 3). Ferric Iron (Fe^3+^) is conveyed through the bloodstream by the transferrin (TF) iron transporter, forming TF-Fe^3+^ (Wallace 2016). The TF-Fe^3+^ complex is absorbed into the cell through endocytosis, following its binding to the transferrin receptor 1 (TFR1). Fe^3+^ then detaches from TF and is converted to ferrous iron (Fe^2+^) by six-transmembrane epithelial antigen of prostate 3 (STEAP3) (Sendamarai et al. 2008). Subsequently, the Fe^2+^ is either stored as ferritin or transferred to the labile iron pool (LIP) via divalent metal transporter 1 (DMT1) (Wang et al. 2021). Ferritinophagy, the process of transporting ferritin to autophagosomes for degradation to release free iron, is uniquely facilitated by nuclear receptor co-activator 4 (NCOA4) (Mancias et al. 2014). When there is an excess accumulation of Fe^2+^, it can instigate lipid peroxidation through both the Fenton reaction and iron-dependent oxidase, causing potential harm (Galy et al. 2023; Li and Li 2020). In amino acid metabolism, extracellular cystine and intracellular glutamate are transported through System Xc‑ on the cell membrane at a ratio of 1:1. Cystine generates glutathione (GSH) through a series of enzymatic reactions (Parker et al. 2021). Reduced GSH has free radical scavenging and antioxidant properties and can effectively reduce the accumulation of reactive oxygen species (ROS). Glutathione peroxidase 4 (GPX4) is a critical molecule in maintaining the reducibility of GSH (Dixon et al. 2012; Yang et al. 2014). Ultimately, the build-up of lipid peroxidation is a central aspect of ferroptosis, wherein polyunsaturated fatty acids (PUFAs) have a significant function. An enzyme known as acyl-CoA synthetase long-chain family member 4 (ACSL4), which is integral to phospholipid metabolism, converts PUFAs to PUFA-CoAs. PUFA-CoAs undergo esterification by lysophosphatidylcholine acyltransferase 3 (LPCAT3) to become PL-PUFAs, which are oxidized by lipoxygenases (LOXs) to form lipid hydroperoxides. The existence of Fe^2+^ allows these lipid hydroperoxides to transform into harmful lipid radicals like alkoxy radicals. This process results in cellular damage and eventually leads to ferroptosis (Dixon and Pratt 2023).

### Ferroptosis role in AIDN

The regulatory mechanisms of ferroptosis-associated genes in AIDN progression are shown in Table 1. Ferroptosis is marked by the buildup of iron-dependent lipid peroxidation within cells and plays a significant role in the onset and progression of diseases related to the nervous system. (Li et al. 2024; Liu et al. 2023b; Qu et al. 2022; Zhang et al. 2023a). Iron, a widely distributed trace element within the human body, assumes crucial functions in diverse facets of brain development and physiology, neurotransmitter synthesis, cytoplasmic protein functionality, and mitochondrial reactions (Singh et al. 2014). Evidence of iron metabolism disorders has also been found in anesthesia-induced developmental neurotoxicity. Wu et al. (Wu et al. 2020) reported that sevoflurane, an inhalation anesthetic, and ketamine, an intravenous anesthetic, led to iron overload and ferroptosis in primary hippocampal neurons cultured in vitro and in neonatal and aged rats in vivo, thereby causing neurotoxicity and cognitive impairment. Most general anesthetics act on NMDA receptors as one of their primary targets (Maksimovic et al. 2022). The study further revealed that ketamine or sevoflurane exposure amplified N-methyl-D-aspartate receptor (NMDAR) subunits. The NMDAR upregulation activated ras-related dexamethasone-induced 1 (RASD1, also named Dexras1, a new GTPase), which could bind to the peripheral benzodiazepine receptor-associated protein 7 (PAP7) and DMT1 to form a ternary complex, thereby enhancing the NMDAR capacity to activate DMT1 for iron absorption. Notably, Deferiprone (DFP) is a small molecule iron chelator that is in clinical use for treating iron overload, markedly in patients with hemosiderosis. In ketamine or sevoflurane-induced neurotoxicity models, DFP pretreatment successfully ameliorated mitochondrial dysfunction, iron death, and cognitive impairment. The findings of this study offer substantial evidence that inhalational and intravenous general anesthetics, like sevoflurane and ketamine, can cause iron accumulation in both developing and elderly brains. This iron overload can lead to cognitive impairment, potentially through neuronal ferroptosis. These findings extend our insight into the mechanisms underlying neurotoxicity induced by general anesthetics, specifically by highlighting the implications for iron metabolism in the brain. Additionally, this research suggests a novel therapeutic approach involving the local depletion of iron or limitation of iron influx, which may prove advantageous in treating neurodevelopmental or neurodegenerative toxicity caused by general anesthesia (GA) or iron overload.

Furthermore, the deleterious effects of repeated exposure to sevoflurane-induced oxidative stress in neonatal mice were similarly demonstrated by Zhang et al.’s study (Zhang et al. 2022a). These findings demonstrated disruptions in the mitochondrial respiratory chain and calcium homeostasis, impairments in mitochondrial membrane permeability and potential, and subsequent induction of iron metabolism disorders. These variations led to neuronal impairment, ultimately resulting in cognitive deficits. However, the study also highlighted that the improvement of mitochondrial function and the effective chelation of neurotoxic iron successfully reverted these pathological alterations.

Building on these insights, a recent study (Kang et al. 2024) underscored the potential role of endoplasmic reticulum (ER) stress in the development of AIDN. The research showed that exposure to sevoflurane led to ferroptosis in hippocampal neurons in the developing brain, a process driven by iron-dependent lipid peroxidation due to the accumulation of iron and hydrogen peroxide (H_2_O_2_). Mechanistically, The exposure to sevoflurane activated the ER stress protein kinase RNA-like ER kinase (PERK)/activating transcription factor 4 (ATF4) pathway, which resulted in the activation of activating transcription factor 3 (ATF3). ATF3, part of the ATF/ CREB (cAMP response element-binding protein) transcription factor family, functions as a stress response factor (Hunt et al. 2012). Its activation led to an increase in H_2_O_2_ in neuronal cells by upregulating NADPH oxidase 4 (NOX4) and inhibiting catalase while also causing a decrease in H_2_O_2_ clearance by inhibiting GPX4 and solute carrier family 7 member 11 (SLC7A11). This resulted in H_2_O_2_ accumulation in neuronal cells. The excessive H_2_O_2_ promoted the upregulation of TFR and TF and the downregulation of ferroportin (FPN) in neuronal cells, which subsequently led to an increase in Fe^2+^ and initiated the ferroptosis cascade process, ultimately causing ferroptosis of hippocampal neurons. The study also found that neuronal death could be mitigated with pretreatment with an ER stress inhibitor, a PERK inhibitor, or siRNA targeting ATF4 or ATF3. This indicates that the activation of ATF3 mediated by PERK/ATF4 due to ER stress is involved in neuronal cell death caused by sevoflurane. The study illuminates the mechanisms behind developmental neurotoxicity caused by sevoflurane, while also unveiling an innovative therapeutic approach to counteract ferroptosis-related neuronal damage in infants and newborns exposed to volatile anesthesia.

Given that fetal and preterm brains are more susceptible to lipid peroxidation and oxidative stress-related cell death than full-term and adult brains (Lafemina et al. 2006; Zhao et al. 2016). Song et al. (Song et al. 2022) discovered that maternal exposure to sevoflurane markedly decreased proliferation and differentiation within the embryonic prefrontal cortex (PFC) and triggered an elevation in ferroptosis within the fetal PFC. The PFC, responsible for regulating emotions and executive functions, contains neural stem cells that can proliferate and generate additional neural precursors. These precursors can differentiate into neurons, astrocytes, or oligodendrocytes (Sargin et al. 2019). Additionally, the research discovered a noteworthy reduction in Nrf2 protein level, coupled with a significant rise in ROS generation in the fetal PFC after maternal exposure to sevoflurane. Nrf2 is a pivotal transcription factor that governs the balance between neurogenesis and ferroptosis (Kahroba et al. 2021). It impacts neural stem cells by enhancing their proliferation and differentiation. Furthermore, it regulates cellular ferroptosis by managing genes that inhibit oxidative stress and decrease lipid peroxidation (Dodson et al. 2019). These findings indicate that Nrf2 is a key mediator in the suppression of the fetal prefrontal cortex’s neurogenesis and ferroptosis induction after maternal sevoflurane exposure. A recent study (Jiang et al. 2023b) also indicated that sevoflurane exposure in midgestational rats resulted in lipid peroxidation and iron accumulation in the fetal rat brain. This triggered ferroptosis in neural cells, impacting the cognitive abilities of the offspring. The mechanism underlying this phenomenon involved the hyperactivation of ataxia telangiectasia mutated (ATM) and its downstream p53/SAT1 (spermidine/spermine N^1^- acetyltransferase 1) pathway, thereby enhancing 15-lipoxygenase 2 (15LO2)-phosphatidylethanolamine binding protein 1 (PEBP1) interaction in fetal brains. Moreover, the use of ferrostatin-1 (a ferroptosis inhibitor), PD146176 (a specific 15LOX inhibitor), or Ku55933 (an ATM inhibitor) could rescue the sevoflurane-induced ferroptosis and cognitive impairment, suggesting that targeted ferroptosis inhibition and its associated targets could have protective effects on sevoflurane induced neurotoxicity. This study recommends weighing the pros and cons of non-obstetric surgery during pregnancy and shows sevoflurane neurotoxicity in the embryonic brain.

Lastly, despite proven neurotoxicity to the developing brain, isoflurane continues to be widely used as a volatile anesthetic worldwide. Based on the findings of Xia et al. (Xia et al. 2018), the study observed that isoflurane caused ferroptosis in primary cortical neuronal cells in vitro through GPX4 suppression, resulting in an increase in ROS production, disruption of the mitochondrial membrane potential, and subsequent cell death. Nevertheless, the administration of the selective ferroptosis blocker, ferrostatin-1, as a pretreatment was discovered to effectively safeguard mitochondrial functionality and mitigate neuronal cell death induced by isoflurane exposure. These findings suggest that ferroptosis plays an important role in isoflurane-induced neurotoxicity and that ferrostatin-1 pretreatment for neuroprotection could be a viable clinical intervention. Nevertheless, Liu et al. (Liu et al. 2021) assessed the role of isoflurane exposure on GPX4 in the hippocampal tissue of developing mice in vivo. Differently, GPX4 levels did not change in hippocampal tissue after isoflurane exposure, whereas SLC7A11 protein, a key factor of cystine/glutamate antiporter (Systemxc-), and its downstream GSH decreased after isoflurane exposure, depending on dose and time. Moreover, isoflurane increased cytochrome c oxidase/complex IV activity in the mitochondrial electron transport chain (ETC), potentially contributing to ferroptosis in hippocampus. Notably, the isoflurane-induced ferroptosis and associated cognitive impairment were effectively reduced by pretreatment with a ferroptosis blocker (ferrostatin-1) or mitochondrial protector (dimethyl fumarate). These findings highlight the interconnectedness between ferroptosis, mitochondria, and isoflurane and offer a unique perspective on therapeutic approaches to counteract isoflurane-induced learning and memory deficits.

## Parthanatos

### The definition and mechanism of parthanatos

Parthanatos was originally discovered in stroke brain nerve cells by Ted Dawson and Valina Dawson couples (Andrabi et al. 2006; Yu et al. 2006; Yu et al. 2002) at Johns Hopkins University School of Medicine, and it has now been officially recognized by NCCD (Galluzzi et al. 2018, 2012). Parthanatos is a regulatory process. Morphologically, parthanatos causes mitochondrial depolarization and plasma membrane disruption without membrane blebbing or swelling (Huang et al. 2022).

**Fig. 4:**
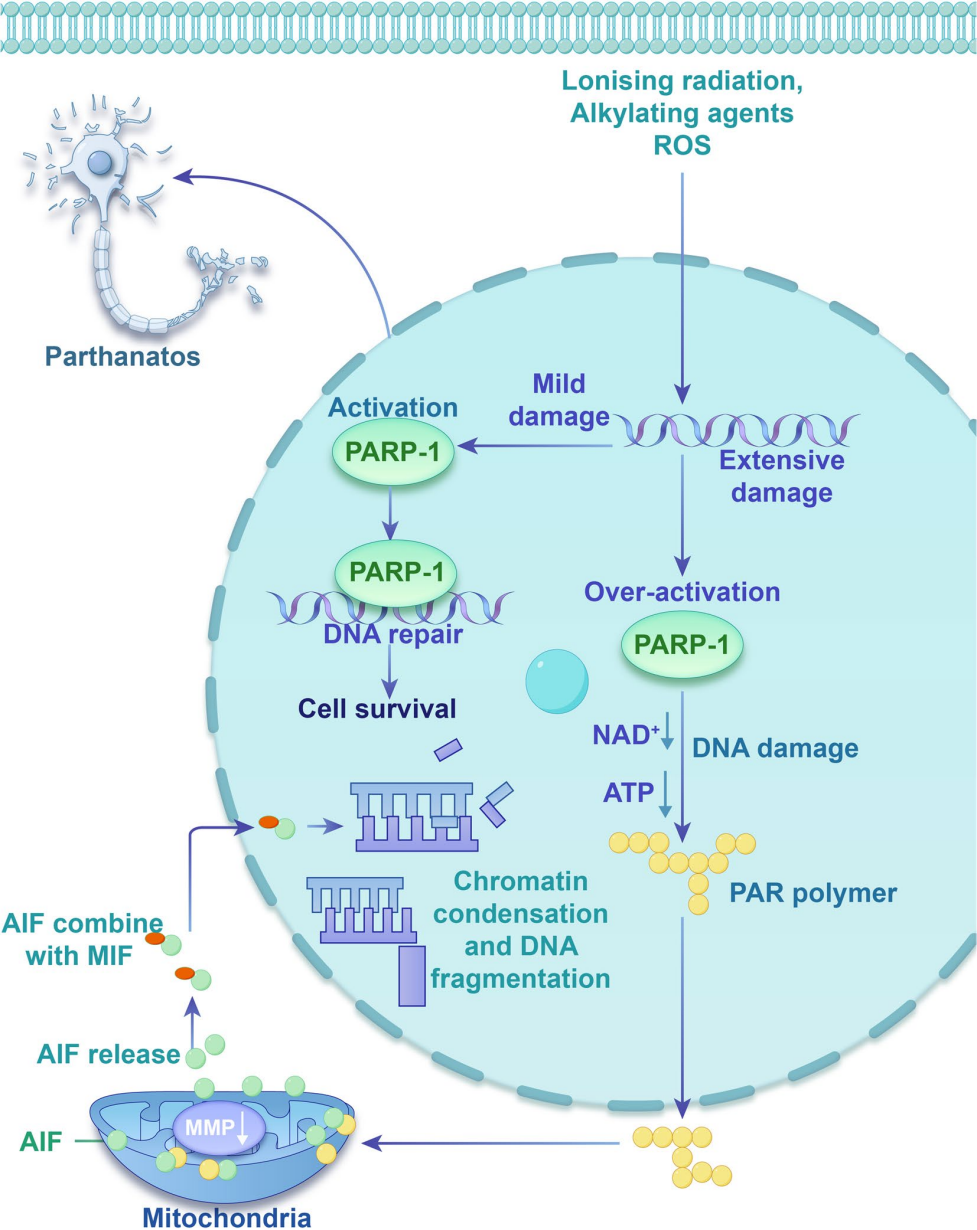
The mechanism of parthanatos

The mechanisms regulating this process have been partially identified (Fig. 4). Poly (ADP-Ribose) polymerase 1 (PARP-1), an ADP-ribosyltransferase, facilitates the transmission of ADP-ribose from nicotinamide adenine dinucleotide (NAD +) to target proteins (Hegedűs and Virág 2014; Zhou et al. 2021). The PARP-1 role was initially elucidated in oncological diseases (Masutani et al. 2003), and PARP-1, which accounts for more than 90% of intracellular PARP activity, is a DNA repair enzyme found predominantly in the nucleus of eukaryotic cells. The classical pathway for PARP-1 activation is through DNA damage. Common causes of DNA fragmentation include ROS, ionizing radiation, and alkylating agents (Liu et al. 2022b; Ma et al. 2016; Pan et al. 2020). PARP-1 activation is related to the level of DNA damage (Wang et al. 2019). PARP-1 repairs DNA damage under normal physiological conditions. Nonetheless, when DNA is significantly compromised, excessive PARP-1 activation leads to the accumulation of poly (ADP-ribose) (PAR), which necessitates large amounts of NAD + . NAD + not only serves as a substrate for PAR synthesis but also acts as a cofactor in several redox reactions, including those in the pentose phosphate pathway, glycolysis, and the tricarboxylic acid cycle (Xie et al. 2020). Furthermore, the PAR translocation from the nucleus to the mitochondria results in the discharge of apoptosis-inducing factor (AIF). Once AIF exits the mitochondria, it combines with macrophage migration inhibitory factor (MIF) in the cytoplasm. The AIF/MIF complex then moves to the nucleus, where it triggers chromatin condensation and DNA fragmentation, ultimately resulting in cell death (Jiang et al. 2023c; Martínez-Morcillo et al. 2021).

### Parthanatos role in AIDN

The regulatory mechanisms of parthanatos-associated genes in AIDN progression are shown in Table 1. Increasing evidence indicates that parthanatos plays a key role in the evolution of various neurological disorders (Jiang et al. 2023a; Wang et al. 2022d; Zhao et al. 2021). Neuronal cell death serves as a crucial mechanism in AIDN. Past studies have demonstrated that anesthetics can instigate various forms of cell death, encompassing apoptosis, pyroptosis, necroptosis, and ferroptosis (Shu and Du 2022; Wu et al. 2020; Xu et al. 2022; Zhang et al. 2022b). However, a specific study by Piao et al. (Piao et al. 2020) established the role of parthanatos in neonatal neuronal cell death caused by sevoflurane exposure. This study revealed that sevoflurane exposure induced parthanatos in various cell types, such as HT22 cells, SH-SY5Y cells, primary hippocampal neurons, and neonatal rat hippocampal tissue. The accumulation of cytoplasmic PAR polymers and AIF translocation to the nucleus as a result of PAPR-1 hyperactivation were the causes of this cell death process. These results comply with parthanatos criterion. Additionally, Sevoflurane exposure caused neuronal DNA damage and increased intracellular ROS production. On the other hand, preventing intracellular ROS reduced DNA deterioration and prevented sevoflurane-induced neonatal neuronal parthanatos. Hence, the findings of this study imply that DNA damage is caused by the excessive generation of ROS within cells as a result of sevoflurane exposure, and this damage is a critical step in the process leading to parthanatos-mediated cell death. This demonstrates a connection between parthanatos and the loss of neural cells in the developing brain caused by sevoflurane.

Furthermore, another study discovered that after exposure to sevoflurane, mid-gestation rats with overactivated PARP-1 experienced neuronal cell death and impaired neurite development. These results were corroborated by Wang et al. (Wang et al. 2022a), who demonstrated that in the fetal rat brain, exposure to sevoflurane significantly reduced neurite formation and caused neuronal cell death. They noticed PARP-1 hyperactivation, cytoplasmic PAR polymer buildup, and nuclear translocation of AIF, which are indicators of parthanatos. Moreover, exposure to sevoflurane caused neurotoxicity linked to an increased release of ROS within cells and an activation of the proline-rich tyrosine kinase 2 (Pyk2)/striatal-enriched phosphatase 61 (STEP61) pathway. Brain tyrosine phosphatase STEP61, which is found in the postsynaptic terminal and endoplasmic reticulum, is linked to a number of pathophysiological processes and neurological conditions (Carvajal and Cerpa 2021; Kurup et al. 2015). Furthermore, Pyk2, a STEP61 substrate, is linked to memory trace formation and synaptic plasticity (Mastrolia et al. 2021).The inhibition of PARP-1 or STEP61 attenuated the cognitive deficits caused by sevoflurane exposure and lessened the suppression of neurite development and neuronal cell death. Collectively, these findings support that sevoflurane exposure during the second trimester of pregnancy can lead to neurotoxicity in rat offspring by hyperactivating PARP1 through the STEP61/Pyk2 pathway. However, research on the role of parthanatos in the regulation of anesthesia-induced developmental neurotoxicity is limited. It has been proven that parthanatos is also implicated in AIDN and its regulation can potentially intervene in the AIDN progression. Therefore, elucidating the specific mechanism of parthanatos and its upstream and downstream regulatory mechanisms is expected to shed new light on AIDN prevention.

## Conclusions and perspective

**Fig. 5:**
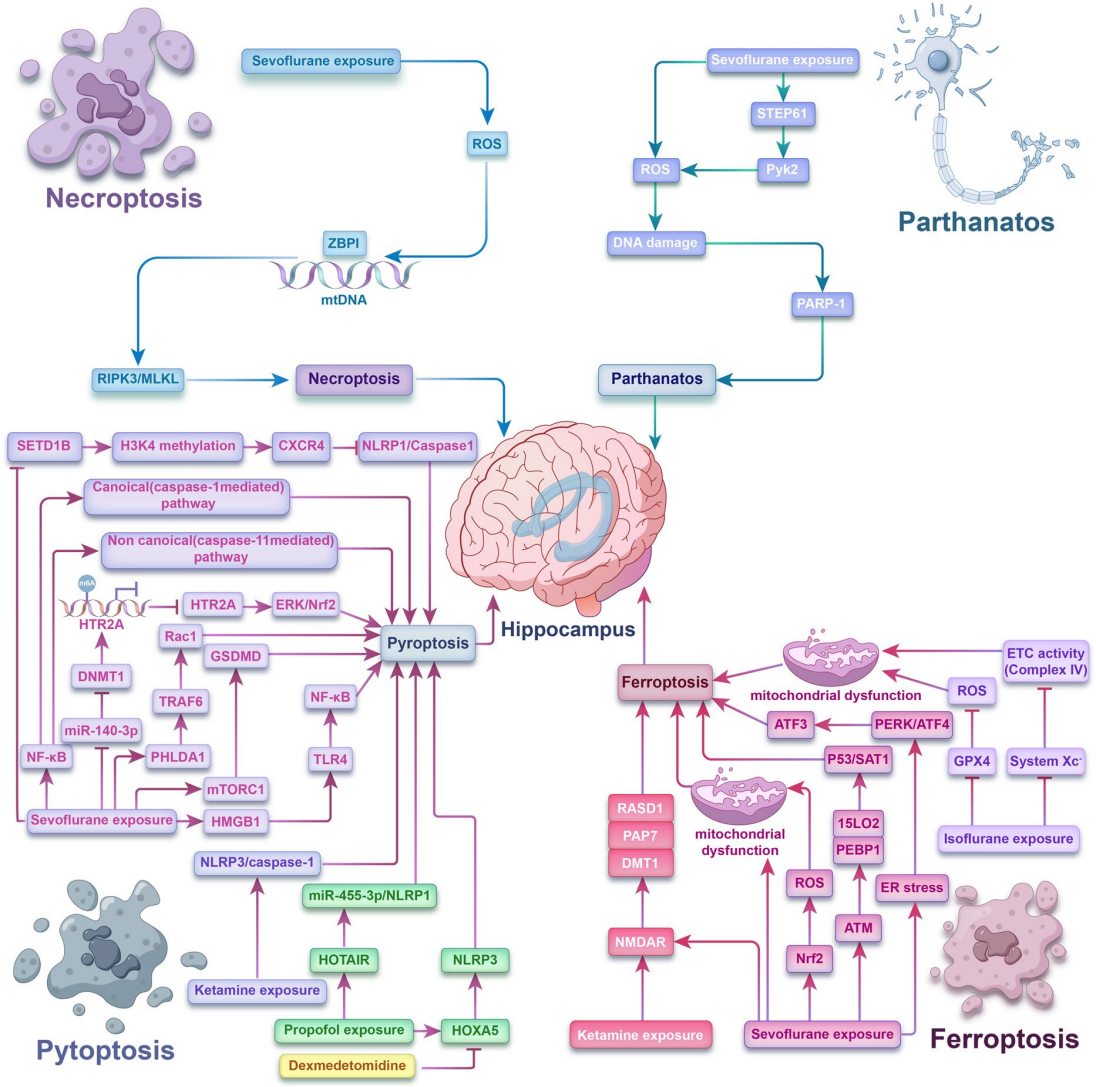
Core molecular mechanisms of non-apoptotic PCDs in AIDN

To date, anesthesia-induced developmental neurotoxicity continues to be a contentious and prominent subject of clinical interest. Despite extensive preclinical investigations, the mechanisms underlying AIDN remain largely elusive. Within the nervous system, the equilibrium between cellular survival and demise influences the normal maturation of the nervous system and the onset and progression of neurological disorders. Through the exploration of various emerging modes of programmed cell death, including necroptosis, pyroptosis, ferroptosis, and parthanatos, these distinct forms of cellular demise exhibit significant functions and contributions to AIDN. In this review, we summarize the core mechanisms of emerging forms of non-apoptotic PCDs in AIDN (Fig. 5). We conclude that (a) non-apoptotic PCDs, like necroptosis, ferroptosis, pyroptosis, and parthanatos, are commonly implicated in AIDN; however, the molecular mechanisms require further exploration. (b) Limited experimental evidence has established a pathological correlation between non-apoptotic PCDs and AIDN, and this warrants further exploration. (c) Quantification of molecular markers of non-apoptotic PCDs in patients diagnosed with AIDN holds potential as a tool for its prevention and treatment.

Based on these findings, future studies should focus on the following areas: (a) conducting a comprehensive investigation into the interrelation among various types of PCDs in AIDN to ascertain the predominant type; (b) exploring the associations between PCDs and other underlying molecular mechanisms in AIDN; (c) investigating the regulatory mechanisms of PCDs in AIDN, particularly those pertaining to the modulation of gene expression levels associated with PCDs; (d) identifying novel agents that target specific PCDs pathways in AIDN for future investigations.

Although non-apoptotic PCDs are critical to AIDN pathogenesis, it is important to emphasize that AIDN complexity cannot be simplified to a single pathophysiological mechanism or the inhibition or activation of a solitary cell death type. Consequently, implementing specific combination therapies targeting multiple PCDs modalities may present a more promising approach for preventing and treating AIDN. Despite the abundance of successful therapeutic interventions demonstrated in animal models of AIDN, the observed benefits at the preclinical stage have largely failed to translate into clinical efficacy. Consequently, the utilization of novel animal models may be necessary to characterize the intricate events underlying AIDN more comprehensively. To conclude, this review offers an in-depth understanding of the various roles of non-apoptotic PCDs in the pathological processes of AIDN. The role of PCDs must be carefully considered and analyzed to improve our understanding of AIDN with uncertain etiology in the future.

## Data Availability

No datasets were generated or analysed during the current study.

## Abbreviations

AIDN: Anesthetic-induced developmental neurotoxicity
PCD: Programmed cell death
RIPK1: Receptor-interacting serine/threonine protein kinase 1
RIPK3: Receptor-interacting serine/threonine protein kinase 3
MLKL: Mixed lineage kinase domain-like protein
TNF-α: Tumor necrosis factor-α
TNF-R1: Tumor necrosis factor receptor 1
TRAF2/5: Tumor necrosis factor (TNF) receptor-associated factor 2/5
TRADD: Tumour necrosis factor receptor type 1 (TNFR1)-associated death domain
LUBAC: Linear ubiquitin chain assembly complex
cIAP1/2: Cellular inhibitor of apoptosis protein 1 and 2
CYLD: Cylindromatosis
TAK1: Transforming growth factor-β-activated kinase 1
TAB2/3: TAK1-binding proteins 2/3
MAPK: Mitogen-activated protein kinase
NF-κB: Nuclear factor-kappaB
FADD: Fas-associated death domain protein
RHIM: Receptor-interacting protein homotypic interaction motif
Nec-1: Necrostatin-1
ZBP1: Z-DNA/RNA-binding protein 1
mtDNA: Mitochondrial DNA,
NCCD: Nomenclature Committee on Cell Death
NLRP1: NOD-like receptor protein 1
NLRP3: NOD-like receptor protein 3
NLRC4: NOD-like receptor C4
AIM2: Absent in melanoma 2
ASC: Apoptosis-associated speck-like protein
CARD: Caspase activation and recruitment domain
PRR: Pattern recognition receptors
GSDMD: Gasdermin D
GSDMD-NT: Gasdermin D-N-terminal
GSDMD-CT: Gasdermin D-C-terminal
Pro-IL-1β: Pro-interleukin-1β
Pro-IL-18: Pro-interleukin-18
IL-18: Interleukin-18
ATP: Adenosine triphosphate
P2X7: Purinergic ligand-gated ion channel 7
GSDME: Gasdermin E
GSDME-NTs: Gasdermin E-N-terminals
TLR: Toll-like receptor
GSDMB-NT: Gasdermin B-N-terminal
GSDMB: Gasdermin B
HTR2A: 5-Hydroxytryptamine receptor 2A
BCL-2: B cell lymphoma 2
DNMT1: DNA methyltransferase-1
ERK: Extracellular-signal-regulated kinase
Nrf2: Nuclear factor erythroid 2-related factor 2
SETD1B: SET domain containing 1b
CXCR4: C-X-C motif chemokine receptor 4
H3K4: Histone 3 lysine 4
PHLDA1: Pleckstrin homology-like domain family A member 1
TRAF6: Tumor necrosis factor receptor associated factor-6
Rac1: Ras-related C3 botulinum toxin substrate 1
mTORC1: Mammalian target of rapamycin complex 1
mTORC2: Mammalian target of rapamycin complex 2
mTOR: Mammalian target of rapamycin
DAMPs: Damage-associated molecular patterns
PMR: Plasma membrane rupture
DSF: Disulfiram
NSA: Necrosulfonamide
HMGB1: High-mobility group box 1
PI3K: Phosphatidylinositol 3-kinase
AKT: Protein kinase B (AKT)
NMDA: N-methyl-D-aspartate
MCC950: An NLRP3 blocker
VX-765: A caspase-1 blocker
HOTAIR: HOX transcript antisense RNA
HOXA5: Homeobox A5
ROS: Reactive oxygen species
RSL3: RAS-selective lethal 3
TF: Transferrin,
Fe^3+^: Ferric Iron,
TFR1: Transferrin receptor 1,
STEAP3: Six-transmembrane antigen of prostate 3,
Fe2 +: Ferrous iron,
DMT1: Divalent metal transporter 1,
LIP: Labile iron pool,
NCOA4: Nuclear receptor co-activator 4,
PUFAs: Polyunsaturated fatty acids,
ACSL4: Acyl-CoA synthetase long-chain family member 4,
LPCAT3: Lysophosphatidylcholine acyltransferase 3,
PL-PUFAs: Phospholipid-bound polyunsaturated fatty acids,
LOXs: Lipoxygenases,
GSH: Glutathione,
GPX4: Glutathione peroxidase 4,
NMDAR: N-methyl-D-aspartate receptor
RASD1: Ras-related dexamethasone-induced 1
PAP7: Peripheral benzodiazepine receptor-associated protein 7
DFP: Deferiprone
GA: General anesthesia
H_2_O_2_: Hydrogen peroxide
ER: Endoplasmic reticulum
PERK: Protein kinase RNA-like ER kinase
ATF4: Activating transcription factor 4
ATF3: Activating transcription factor 3
CREB: CAMP response element-binding protein
NOX4: NADPH oxidase 4
SLC7A11: Solute carrier family 7 member 11
FPN: Ferroportin
PFC: Prefrontal cortex
ATM: Ataxia telangiectasia mutated
15LO2: 15-Lipoxygenase 2
PEBP1: Phosphatidylethanolamine binding protein 1
SAT1: Spermidine/spermine N^1^- acetyltransferase 1
Ferrostatin-1: A ferroptosis inhibitor
PD146176: A specific 15LOX inhibitor
Ku55933: An ATM inhibitor
ETC: Electron transport chain
PARP-1: Poly (ADP-ribose) polymerase 1
NAD +: Nicotinamide adenine dinucleotide
PAR: Poly (ADP-ribose)
AIF: Apoptosis-inducing factor
MIF: Migration inhibitory factor
Pyk2: Proline-rich tyrosine kinase 2
STEP61: Striatal-enriched phosphatase 61
